# Vocational rehabilitation job coaching based on the Individual Placement and Support model: protocol for a mixed methods implementation study

**DOI:** 10.3389/fresc.2026.1833369

**Published:** 2026-07-01

**Authors:** Riitta Seppänen-Järvelä, Maarit Karhula, Hennariikka Heinijoki

**Affiliations:** Social Insurance Institution of Finland, Helsinki, Finland

**Keywords:** implementation, implementation science, Individual Placement and Support (IPS), rehabilitation, vocational rehabilitaion

## Abstract

This protocol outlines how vocational rehabilitation job coaching based on the Individual Placement and Support (IPS) model is examined using a mixed methods implementation study design. Job coaching targets working-age individuals whose ability to work has been substantially reduced. The goal of this study is to examine job coaching from the following perspectives: client outcomes, service outcomes, implementation outcomes and determinants, and the processes underlying its embedding in practice. Survey and interview data will be collected from different stakeholder groups: clients, job coaches, supervisors, and rehabilitation claims processors. Employing mixed methods and multiple data sources provides a comprehensive understanding of the contextual factors, implementation processes, and perceived meanings associated with this complex vocational rehabilitation intervention. The study is grounded in health services research in rehabilitation and in implementation science to understand how job coaching is conducted and implemented in order to inform decisions about rehabilitation policy and practice. This study addresses a timely need to examine the implementation of evidence-based vocational rehabilitation and to generate knowledge on the application of job coaching to a new target group.

**Clinical Trial Registration:**

https://doi.org/10.17605/OSF.IO/N63H5.

## 1 Introduction

Supported Employment (SE) aims to promote the employment of individuals in disadvantaged labor market positions, such as the long-term unemployed, persons with disabilities, or individuals with mental health conditions, in the open labor market. The Individual Placement and Support (IPS) model is the best-known and most extensively studied evidence-based form of SE (1–3). IPS is an individualized service because its core principles and practices are built around the unique goals, needs, and strengths of each person. The entire process is guided by the person’s strengths and preferences.

In Finland, SE, including job coaching, has been relatively well established, although services have been heterogeneous in terms of providers, objectives, methods, and target groups (4). Efforts to promote the adoption and adaptation of the IPS model in the Finnish context began nearly a decade ago, led by various actors (5, 6). The nationwide rollout of IPS job coaching was initiated under the National Mental Health Strategy (2020–2030), accompanied by an evaluation study (7, 8). At the beginning of 2025, the Social Insurance Institution of Finland (Kela) launched job coaching, a form of vocational rehabilitation based on the IPS model.

The IPS model is grounded in the principle of rapid job search, whereby clients seek positions directly in the open labor market (9). Clients receive individualized, needs-based support that continues during employment (2). Time and ﬂexibility are crucial to the development of a helpful relationship with the employment specialist (10). Integration with other services is essential, as employment support is combined with health and social services (11). Close collaboration with employers is another core principle and a critical factor for successful implementation (12).

The effectiveness of IPS has been demonstrated particularly among people with mental health conditions (13): employment rates are up to twice as high compared to traditional vocational services (14). Furthermore, IPS is more effective and potentially cost-effective than traditional vocational rehabilitation in people with severe mental illness receiving sickness or disability benefits (15). It has also been shown to improve participants’ self-esteem and life-satisfaction (16). The confidence and vocational competence gained during training may support longer-term employment relationships (17). Nevertheless, despite the high employment rates, many jobs obtained through IPS are short-term, generating pressure for continuous support provision (18). However, work achieved via supported employment, including IPS, provides the possibility of gaining significance and recognition (19). It is often low-paid and unstable, offering limited prospects for sustainable careers, and, as a result, clients frequently continue to rely on social security benefits (20).

Whereas IPS has primarily been studied among people with mental health conditions (13), research and adaptations have expanded to other populations (21), such as veterans with post-traumatic stress disorder (22), persons with physical disabilities, for example spinal cord injury (23) or chronic pain conditions (24), those with neurocognitive impairments, such as autism spectrum disorder (25) or substance use disorders (26, 27), and the long-term unemployed (28). Core IPS principles—such as individualization and rapid job placement—have been found applicable in a variety of disadvantaged labor market contexts. However, adaptation to the specific needs of each target group is essential (1, 2, 11, 30). Without integrated support, the effects of IPS may remain modest, particularly among the long-term unemployed and socially marginalized populations (28, 29). Metcalfe and Drake (30) note that as IPS expands to serve new populations, it will be important to document and understand the links between individual characteristics, variance in participation patterns, and employment outcomes.

While SE and the IPS model have been shown to be effective and encouraging results have been obtained from adapting the model for different target groups, there remains a recognized need to pay particular attention to implementation-related issues (29, 31). In the implementation of IPS, emphasis is placed on fidelity to evidence-based principles and on assessing fidelity using standardized measures (2).

A review of the barriers and facilitators of IPS implementation found that evaluation studies have often applied varying implementation concepts and frequently lacked a strong theoretical foundation (31). Recent studies have explored, for example, accessing the IPS service through a zero-exclusion approach (32) the role of external contextual factors in implementation (33, 34), the impact of implementation strategies (18), turnover of employment specialists (35) and the sustainability and quality of employment as well as the employer’s perspective (20). Overall, further research is needed on the different phases and perspectives of adoption and implementation (36), as well as on the interaction between facilitating and hindering factors (31).

This protocol outlines how job coaching, an IPS-based form of vocational rehabilitation, is examined using a mixed methods implementation study design. The study addresses a timely research interest at the implementation of evidence-based vocational rehabilitation producing new knowledge of the intervention to a new target group.

## 2 Methods and analysis

### 2.1 Study context

In Finland, Kela is responsible for the vocational rehabilitation of young people and adults who are not currently integrated into working life. Vocational rehabilitation supports work ability and career continuity of working-age individuals. Kela offers job coaching, carried out by local service providers, as part of their vocational rehabilitation services (37). At the moment in the year 2026, there are 69 service providers delivering Kela’s job coaching around Finland.

Job coaching is a personalized service targeted at individuals of working age whose ability to work has been substantially reduced but whose functional capacity and life situation allow them to work or to be self-employed. Processing of rehabilitation claims requires a medical certificateAccess to vocational rehabilitation for young people (16–29 years old) is without diagnostic criteria (38).

Job coaching is a form of supported employment based on the IPS model (39), adapted to the context of vocational rehabilitation. Its implementation is guided by a service description (40), which defines the features of the intervention. The IPS model was used in the design of the service description. Table 1 illustrates the relationship between Kela’s job coaching and the IPS model from a fidelity perspective.

**Table 1:** Kela’s job coaching compared with IPS fidelity components.

IPS-25-criteria	IPS-fidelity feature (39)	Kela’s job coaching feature (40)	Assessment of comparability^a^
Caseload	≤20 per coach	≤20 per coach	Comparable
Employment focus	Employment services	Role specialized in job coaching	Comparable
Vocational generalist	The same coach throughout all phases	The coach is responsible for the entire process	Comparable
Teamwork	A vocational unit structure, role of emplyment supervisor, frequency of supervision, executive teams support	A team (3–10), a team lead, and regular meetings, support	Comparable
Integration with mental health treatment teams and clinical staff	Integrated into the treatment structure	Systematic network collaboration with social and health services, employment services. Integration is implemented through a networking rather than through an organizationally integrated team	Partially comparable
Zero exclusion	Any motivated client can participate	Target group limited to vocational rehabilitation clients	Not comparable
Rapid job search	First contact ≤30 days	The start of on-the-job work defined; job phase ≤1 month	Comparable
Individualized job search	Client’s preferences guide the search	Vocational profiling and an individualized plan guide the job search	Comparable
Employer contacts	Regular, high-quality contacts	Active employer engagement, no quantitative employer-contact target	Partially comparable
Competitive employment	Open labor market	Open labor market/entrepreneurship	Comparable
Ongoing support	No time limit	≤13 months + limited follow-up meetings	Partially comparable
Fidelity monitoring	Regular IPS-25 reviews	As a service arranger, Kela monitors and evaluates effectiveness indicators, for example, but does not require service providers to use the IPS Fidelity Scale	Not comparable

The IPS-25 criteria and IPS fidelity refer to the 25-item Fidelity Scale used in the IPS model (presented here in a concise form).

a Comparable = identical; partially comparable = adapted to fit the Finnish vocational rehabilitation context; not comparable = component altered.

Job coaching includes a maximum of 25 meetings and 95 rehabilitation days, an average of 5 rehabilitation days per week. Job coaching has three consecutive stages: planning phase for job search (1–3 meetings between a client and a coach), 2) support phase for job search (1–20 meetings between a client and a coach), and 3) work placement phase (up to 95 rehabilitation days).

Kela’s job coaching was designed in line with the principles of IPS; however, two criteria—eligibility and service duration—have been modified (see 40). These modifications are planned adaptations to the Finnish vocational rehabilitation context. In terms of eligibility criteria, job coaching is intended for individuals whose illness has caused a substantial reduction in their ability to work. A physician assesses the individual’s need for rehabilitation and provides a medical statement. Duration of the service is defined up to 95 rehabilitation days and must be completed within 13 months. It must begin no later than 1 month after the client’s first meeting. Two follow-up meetings are included.

### 2.2 Study aims

The present study is grounded in health services research in rehabilitation (41) and in implementation science (42, 43) to understand how job coaching is conducted and implemented in order to inform decisions about rehabilitation policy and practice. The study employs a theory-oriented, multi-perspective, mixed methods design (44, 45).

The goal of this study is to examine job coaching from the following perspectives: client outcomes, service outcomes, implementation outcomes and determinants, and the processes underlying its embedding in practice, through the following aims:

#### 2.2.1 Aim 1: client outcomes

- Employment: The types of jobs and employment contracts the clients obtain.
- Job matching: How well the jobs and employment contracts clients obtain match their skills and expectations
- Work engagement and meaningful work: Expectations and experiences related to work engagement, including perceptions of meaningful work and the support received to engage in work.

#### 2.2.2 Aim 2: service outcomes

- Timeliness: The appropriateness of timing of job coaching.
- Client-centeredness: Consideration of the client’s needs, values and wishes.
- Effective: Providing job coaching to those who are likely to benefit from it.

#### 2.2.3 Aim 3: implementation outcomes and determinants explaining the implementation context

- Acceptability: Perceived coherence and making sense of the intervention.
- Appropriateness: Perceived fit of job coaching to the target group and the vocational rehabilitation context, including workplaces.
- Feasibility: Delivery of job coaching by service providers in real-world settings.
- Fidelity and adaptability: Delivery of job coaching in accordance with Kela’s service description and adaptation to the context.
- Determinants explaining the implementation context: Stakeholders’ experiences of factors that facilitate or hinder the delivery and implementation of job coaching.

#### 2.2.4 Aim 4: processes underlying the embedding of job coaching in practice

- Normalization processes: Integration of job coaching into routine practice.
- Factors and mechanisms associated with goals of job coaching: Successful employment and perceived usefulness, and the perceived value of the intervention.

### 2.3 Study design

Personalized work-related rehabilitation processes (46), such as those in job coaching, involve multiple stakeholders who pursue different goals within a multi-actor ecosystem. Applying mixed methods and drawing on multiple data sources enables a comprehensive understanding of the context, processes, and meanings associated with this complex intervention in vocational rehabilitation. The design of this study is based on the interactive, system-based approach, which emphasizes a dynamic system of interrelated components, such as research aims, methods, theories, models, frameworks, and integration. The approach views a mixed methods study as a set of interconnected decisions that influence one another throughout the research process and are emergent and context-sensitive (47).

**Figure 1:**
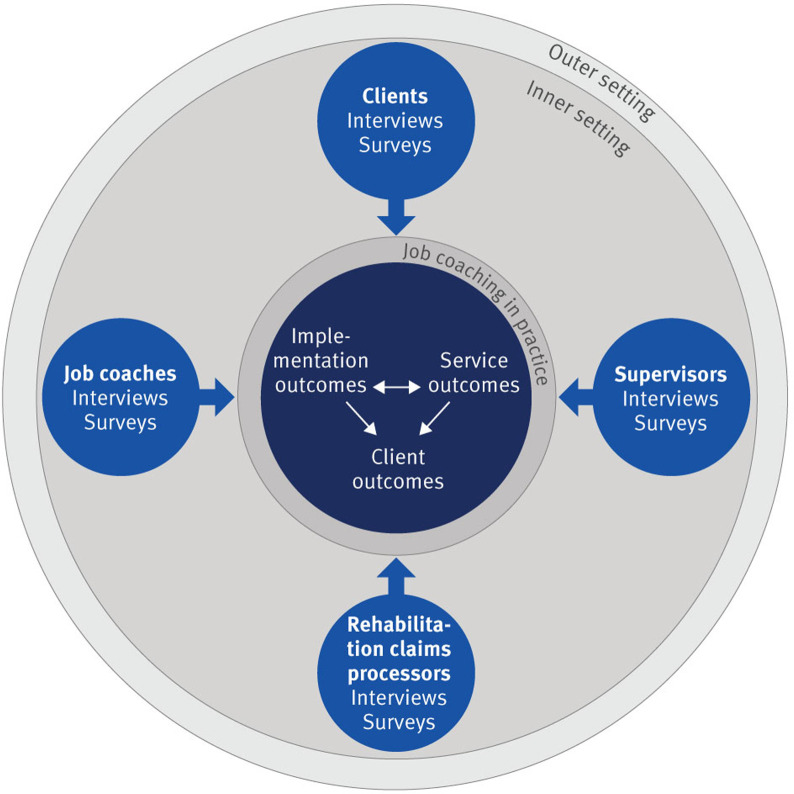
Study design. Solid arrows indicate unidirectional influences and double-headed arrow indicate reciprocal relationship.

The design of this multi-perspective mixed methods implementation study is presented in Figure 1. The study is informed by theories, models, and frameworks (TMFs) used in implementation science (43). This study draws on Proctor et al.’s (48) taxonomy of implementation research targets to guide its overall analytical focus, encompassing the client, service and implementation outcomes, and in addition of Proctor’s category, the processes underlying the embedding of job coaching in practice. In line with this taxonomy, the study addresses multiple analytical aims by examining how the service affects clients, how it is implemented in practice, and which mechanisms and factors shape these outcomes.

To support these aims, the Consolidated Framework for Implementation Research (CFIR), Theoretical Domains Framework (TDF) and Normalization Process Theory (NPT) are used in a complementary and flexible manner across different phases of the study. Using a combination of TMFs−common in implementation research −offers an opportunity to view implementation comprehensively from multiple perspectives and strengthens the validity of the study through theory triangulation (49). Following recent methodological discussions in implementation science (50), these TMFs are treated as heuristic resources rather than fixed analytic templates. That is, they inform the overall orientation of data collection and analysis without being applied deductively as predefined coding structures.

In this study, CFIR and TDF, both adopted within the field of rehabilitation science (51), are used as heuristic resources to help structure the identification and examination of factors associated with implementation outcomes. CFIR (52) provides an overall structure for exploring the broader context of implementing job coaching by bringing together considerations related to five CFIR domains: the implementation process, the innovation, individuals, and the inner and outer settings relevant to implementation outcomes. In this study, the inner setting includes the key actors embedded within the job coaching service, namely clients, job coaches, employers, and rehabilitation claims processors (see Figure 1). The outer setting (i.e., economic, cultural, and political condition) is examined as part of the broader contextual environment when identifying barriers and facilitators to implementation.

TDF is used to complement the CFIR, as it comprehensively captures individual-level psychological and behavioral factors that influence implementation and can inform the analysis of implementation strategies (53). Together, the two frameworks provide a more comprehensive, multi-level understanding of implementation barriers and facilitators, which can contribute to the development of future implementation strategies (54).

NPT (55, 56) is an implementation theory that explain how a complex intervention is implemented and becomes embedded in routine practice. It focuses on the work people do to make an intervention happen in real-world settings. NPT identifies four social mechanisms of normalizing complex practices and the relationships between these: coherence, cognitive participation, collective action, and reflexive monitoring. In this study, NPT is used heuristically to support understanding of how the job coaching service is enacted in practice. It informs the planning of data collection, analysis, and interpretation related to Aim 3–4, which explore the job coaches’ understanding of implementation outcomes and determinants explaining the implementation context and processes underlying the embedding of job coaching in practice.

### 2.4 Data collection

The study employs a mixed methods design, incorporating individual interviews and surveys. Data are collected from multiple stakeholders, including clients, job coaches, supervisors representing employers, and rehabilitation claims processors. Coaches represent the rehabilitation service providers in this study. The study does not combine data at the individual level.

The heuristic approach of applying TMFs introduced by Nilsen et al. (50) is used during the development of the data collection methods (surveys and interview guides). This approach ensures that instrument development is informed by the study aims, which are structured according to Proctor et al. and informed by CFIR, TDF, and NPT, thereby providing conceptual breadth and relevance. However, individual survey items or interview questions are not explicitly mapped onto specific constructs within these frameworks, allowing participants to articulate experiences and meanings that may extend beyond established categories. In addition to these theoretical frameworks, the development of the data collection methods is guided by the service description, particularly its specification of desired client outcomes and its articulation of how the service is intended to be delivered.

The data will be collected in a parallel manner at various stages of the intervention process, providing a comprehensive overview of the implementation of job coaching. Table 2 shows the dataset of the study and sources informing the data collection methods. Table 3 shows how TMFs are linked to the study aims.

**Table 2:** The dataset of the study and source informing the data collection methods.

Method, data source and timeline	Source informed by data collection methods									Type of data	
	SD	CFIR Innovation	CFIR Individuals	CFIR Inner settings	CFIR Outer settings	TDF (DIBQ-mp)	NPT Coherence	NPT Collective action	NPT Reflexive monitoring	QUAN	QUAL
Survey, clients, Oct 1, 2025-Dec 31, 2026	x	x	x	x	x					x	x
Interview, clients, April 1– Sept 25, 2026	x	x	x	x	x						x
Survey, coaches, Mar 16–April 10, 2026	x	x	x	x	x	x	x	x	x	x	x
Interview, coaches, Feb 1–Apr 30, 2026	x	x	x	x	x		x	x	x		x
Survey, supervisors, May 25–Sept 25, 2026	x	x	x							x	x
Interview, supervisors, Apr 13–Oct 30, 2026	x	x	x	x	x		x	x	x		x
Survey, rehabilitation claims processors, Jan 19–Jan 30, 2026	x	x	x							x	x
Interview, rehabilitation claims processors, Feb 9–Feb 28, 2026	x	x	x	x	x						x

CFIR, Consolidated Framework for Implementation Research; DIBQ-mp, Determinants of Implementation Behavior Questionnaire, Finnish version; NPT, Normalization Process Theory; QUAN, quantitative data; QUAL, qualitative data; SD, Kela’s service description; TDF, Theoretical Domains Framework.

**Table 3:** Mapping of TMFs to study aims.

Aim and outcome domain	TMF: CFIR & TDF	TMF: NPT	Stakeholder group: data source
Aim 1: Client outcomes^a^			
Employment/entrepreneurship: The types of jobs and employment contracts the clients obtain.	NA	NA	Cl: S & I; Co: S & I; Su: S & I
Job matching: How well the jobs and employment contracts clients obtain match their skills and expectations.	NA	NA	Cl: I, Co: I
Work engagement and meaningful work: Expectations and experiences related to work engagement, including perceptions of meaningful work and the support received to engage in work.	NA	NA	Cl: S & I
Aim 2: Service outcomes^a^			
Timeliness: The appropriateness of the timing of job coaching.	NA	NA	Cl: S & I, Co: I RCP: S & I
Client-centeredness: Consideration of the client’s needs, values and wishes.	NA	NA	Cl: S & I, Co: S & I
Effective: Providing of job coaching to those who are likely benefit from it.	NA	NA	Co: I, RCP: S & I
Aim 3: Implementation outcomes and determinants explaining the implementation context			
Acceptability^a^: Perceived coherence and making sense of intervention.	CFIR: Innovation, Inner setting, Outer setting, Individuals	Coherence	Cl: S & I, Co: S & I; RCP: S & I
Appropriateness^a^: Perceived fit of job coaching to the target group and the vocational rehabilitation context, including workplaces.	CFIR: Innovation, Inner setting, Outer setting, Individuals	Coherence	Cl: S & I, Co: S & I, Su: S & I, RCP: S & I
Feasibilty^a^: Delivery of job coaching by service providers in real-world settings.	CFIR: Innovation, Inner setting, Outer setting, Individuals	Collective action	Co: S & I
Fidelity and adaptability^a^: Delivery of job in accordance with Kela’s service description and adaptation to the context.	CFIR: Innovation, Inner setting, Outer setting, Individuals	Collective action Reflexive monitoring	Cl: S & I, Co: S & I, Su: S & I
Determinants explaining implementation process and outcomes: Stakeholders’ experiences of factors that facilitate or hinder the delivery and implementation of job coaching.	CFIR: Innovation (TDF: Innovation), Inner setting (TDF: Organization, Innovation strategy, Social influences) Outer setting, Individuals (TDF: Knowledge, Skills, Beliefs about capabilities, Beliefs about consequences, Patient/client, Behavioural regulation)	NA	Cl: S & I, Co: S (including DIBQ-mp) & I, Su: S & I, RCP: S & I
Aim 4: Processes underlying the embedding of job coaching in practice			
Normalization processes: Integration of job coaching into routine practice. Factors and mechanisms associated with goals of job coaching: Successful employment and perceived usefulness, and the perceived value of the intervention.	CFIR: Innovation, Inner setting, Outer setting, Individuals TDF to further specify the CFIR domains Innovation, Inner setting and Individuals	Coherence Collective Action Reflexive Monitoring	Cl: S & I, Co: S (including DIBQ-mp) & I, Su: S & I, RCP: S & I

CFIR, Consolidated Framework for Implementation Research; Cl, client; Co, coaches; DIBQ-mp, Determinants of Implementation Behavior Questionnaire, Finnish version; I, interview; NPT, Normalization Process Theory; RCP, rehabilitation claims processors; S, survey; Su, supervisors; TDF, Theoretical Domains Framework; TMF, theory, model and framework.

a Proctor’s taxonomy (40) was used to organize and target the study aims and outcomes.

#### 2.4.1 Surveys

Data will be collected through electronic surveys from four respondent groups: (1) clients, (2) supervisors, (3) job coaches, and (4) Kela’s rehabilitation claims processors (see Table 1).

The structure of the survey instruments was informed by the job coaching process to align the questionnaire flow with the service trajectory. Thematic domains were organized according to sequential process stages: for example, client surveys begin with entry into the service (e.g., referral), followed by items on job search phases and subsequent support. This structure was expected to facilitate respondents’ orientation and support coherent responses. Survey item development followed the heuristic approach (see Section 2.4 Data collection). Table 2 summarizes which theoretical models/frameworks informed the different surveys. Except for the DIBQ-mp questionnaire (57) used for job coaches, all items were developed by the research team.

An expert review was conducted to ensure content validity and clarity. Initial survey versions were evaluated by three Kela job coaching specialists and one physician, who provided feedback on the relevance and comprehensiveness of items, as well as the clarity and appropriateness of response options. The feedback was discussed with the research group, and the instruments were revised accordingly prior to data collection.

The implementation of electronic surveys utilizes the LimeSurvey application. The LimeSurvey application is maintained by Kela’s information technology (IT) service production within the analytics environment. Responses to the survey are provided anonymously, and the data are stored in such a way that they cannot be linked to individual respondents.

##### 2.4.1.1 Clients

All participants in the job coaching will be invited to complete a survey at the end of rehabilitation, regardless of whether it occurs before or after workplace placement. This will ensure that clients who might drop out before they are employed will be invited to the survey.The target response rate is approximately 60%. Data collection will take place between October 2025 and December 2026. The survey will cover the following areas: work and life situation prior to job coaching, entry into job coaching, job search planning phase, job search support phase, workplace participation phase, and an overall assessment of the job coaching service. If a participant does not complete the service, the survey questionnaire will not include items related to the workplace participation phase.

##### 2.4.1.2 Supervisors

An electronic survey is distributed to all supervisors of job coaching clients. The target response rate is approximately 30%. Reminder emails are sent to encourage participation. Data collection will take place between May 2026 and September 2026. The survey domains include: organization and sector, supervisor’s role in job coaching, recruitment through job coaching, implementation of the job coaching service, and evaluation of the service.

##### 2.4.1.3 Job coaches

An electronic survey is sent to all service providers delivering Kela’s job coaching services. The target response rate is approximately 80%. Reminder emails are sent. Data collection will take place in March 2026. The survey domains include: the job coach’s work and its organization, initiation of the job coaching service, job search planning phase, job search support phase, and workplace participation phase. The Finnish version of the Determinants of Implementation Behavior Questionnaire (DIBQ-mp) will be used as part of the survey to identify factors influencing job coaches’ implementation behavior (57).

##### 2.4.1.4 Kela’s rehabilitation claims processors

An electronic survey is sent to all administrative officers responsible for processing applications for job coaching, estimated at around 130 professionals. Data collection took place in January 2026, with a target response rate of approximately 60%. The survey domains include: target groups and assessment of rehabilitation needs, decision-making process, benefit guidelines and service descriptions, as well as suggestions for development.

#### 2.4.2 Interviews

Thematic interviews are conducted individually with clients, supervisors, and coaches. Interviews are primarily conducted by telephone or via Microsoft Teams; however, participants are offered the option to choose a face-to-face interview if they prefer. Participants are asked to provide consent, which is given verbally and recorded during the interview. The interviews are audio-recorded and subsequently transcribed. The transcribed interviews are pseudonymized.

##### 2.4.2.1 Clients

Interviews are scheduled to take place approximately 2 months after the participant has commenced employment. The aim is to interview 30 individuals between April and September 2026, selected in order of enrollment from various regions across Finland. The interviews explore different phases of job coaching and the support provided during the process, perceived benefits of job coaching, experiences of meaningful work, and work engagement.

##### 2.4.2.2 Supervisors

The aim is to interview 25 workplace supervisors. Interviews will be conducted between April and October 2026. The interviews explore the role of the supervisor and workplace in job coaching, barriers and facilitators in recruiting job coaching clients, and functionality of job coaching.

##### 2.4.2.3 Coaches

The aim is to interview 15 coaches. Interviews will be conducted between February and April 2026. The interviews explore implementation, functionality and benefits, and adaptation of job coaching.

##### 2.4.2.4 Kela’s rehabilitation claims processors

The aim is to interview 6 experts handling applications for job coaching. Interviews were conducted in February 2026. The interviews explore the assessment of the applicant’s overall situation, evaluation of the timeliness and appropriateness of the rehabilitation, sufficiency and quality of the necessary information, and grounds for approval or rejection of the application.

#### 2.4.3 Recruitment of the informants

Voluntary participation and the distinction between rehabilitation and research are clearly explained in the information sheet designed for each stakeholder group and data collection method. In addition, these aspects are underlined in the information sessions targeted at service providers.

Selection bias is managed in several ways: all service providers, clients, supervisors, and rehabilitation claims processors are invited to participate in the study; standardized information sheets, data handling procedures, and recruitment processes are used; and service providers do not select participants. All clients are invited to complete an end-of-rehabilitation survey, regardless of whether they completed the service. For clients who did not complete the service, items related to the workplace participation phase are not included. This ensures that clients who drop out before employment are still invited to participate in the survey.

##### 2.4.3.1 Service providers

All service providers delivering job coaching under Kela are invited to participate in the study. They are participating in their role as contractual partners of Kela and serve both as informants and as recruiters of client and supervisor participants. Participation as an informant in the study is voluntary and confidential. At the launch of the study, all service providers and their coaches were invited to an online information session on August 21, 2025 (via Microsoft Teams). Researchers introduced the study plan, with a particular focus on the dual role of job coaches as informants and as recruiters of both clients and supervisors for the study.

They were introduced to the information materials that they were expected to provide to and go through with clients and supervisors. The material for clients includes information that non-participation or withdrawal will not affect Kela benefits, job coaching services, continuation of support, employment relationships, or workplace accommodations. The recording of the information session and the related materials were distributed to the service providers. A second information session was held in a similar manner on January 13, 2026, focusing on data gathering.

##### 2.4.3.2 Coaches

Coaches were recruited to the study through an invitation presented during the aforementioned information session, which outlined their role as informants. Service providers distribute the link to the electronic survey and accompanying information sheet to coaches working within their organization.

Recruitment for the interviews will proceed as follows: In the survey, coaches may indicate their interest in participating in an interview by granting permission for the researcher to contact them via email. Once a coach has expressed interest and provided consent for contact, the researcher will arrange the interview. If the number of willing participants is high, a purposive sample will be selected to ensure geographic diversity across Finland. The aim is to obtain a varied dataset; thus, data saturation is not a central criterion.

##### 2.4.3.3 Clients

At the final rehabilitation discussion, all clients will be invited to complete an electronic survey. For the interview, participants will be recruited through purposive sampling (58): coaches from different regions of Finland will invite clients to participate. Once the client has been working at the workplace for approximately 2 months, the coach will provide them with an information sheet about the study. Upon expressing interest and granting permission to be contacted, the researcher arranges the interview directly with the client. The goal is to obtain a diverse dataset; therefore, data saturation is not a central criterion.

##### 2.4.3.4 Employers/supervisors

Supervisors represent the employers in this study. The supervisors will be recruited by sending them an information sheet and a link to the electronic survey via email. The supervisors’ email addresses were obtained from the rehabilitation service providers. Recruitment for telephone interviews followed the same procedure: In the survey, supervisors indicated their interest in participating in an interview by granting permission for the researcher to contact them via email. Once a supervisor has expressed interest and provided consent to be contacted, the researcher will arrange the interview.

##### 2.4.3.5 Kela’s rehabilitation claims processors

The study information sheet and survey link are sent via email to all employees in Kela involved in processing job coaching applications. In the survey, the respondents indicated their interest in participating in an interview by granting permission for the researcher to contact them via email. Once the person has expressed interest and provided consent to be contacted, the researcher will arrange the interview.

### 2.5 Data analysis

Data analysis employs mixed-methods integrative strategies, along with a heuristic and abductive approach to applying TMFs (50). Rather than assuming stable or linear pathways, the analysis seeks to identify and explore unexpected relationships and evolving implementation dynamics.

The overall dataset, consisting of both quantitative and qualitative data (see Table 2), is first analyzed using appropriate methods. The analysis strategy allows for either mono or mixed methods to effectively address the study aims. When conducting mixed-methods analysis and applying abductive reasoning, the data integration strategy is described further in this section.

**Qualitative data** from the interviews and open-ended questions from the surveys are imported and analyzed in qualitative data analyzing software Atlas.ti. The data are pseudonymized before importing into the Atlas.ti software. The qualitative analysis process applies thematic analysis as a method for identifying and analyzing patterned meanings within data (59). In the thematic analysis, textual data are segmented into meaning units and iteratively coded. Codes are assigned descriptive labels and systematically grouped, then further developed into themes that capture key patterns and features of the dataset in relation to the aims. Contextualization will be crucial in the coding process: decontextualization breaks data into meaning units removed from their original context, while recontextualization compares these units back to the dataset to ensure that emerging themes reflect the overall meaning (60). The themes developed in inductive analysis will be linked to TMFs (see Table 3). The coding process will be conducted iteratively, with multiple rounds of coding enabling themes to be progressively developed and refined rather than imposed prematurely. Collaborative discussions within the research team will be used to support reflexivity and deepen interpretation.

**The quantitative data** collected through surveys are analyzed using descriptive statistics obtained with IBM SPSS. In the descriptive analyses, the number of respondents for each question will be reported. These analyses will include the examination of central tendencies (e.g., means and medians), dispersion (e.g., standard deviations), and frequency distributions to provide an overall understanding of the data.

Following descriptive analyses, relationships and dependencies between variables will be examined. For example, associations between coaches’ background characteristics (e.g., number of clients, years of work experience) and their perceived ability to deliver job coaching will be examined, as well as associations between clients’ educational and work history and variables related to perceived support received from the coach. Continuous variables will be analyzed using correlation analyses (e.g., Spearman’s rank correlation), while associations between categorical variables will be examined using cross-tabulations and Chi-square or Fisher’s exact tests, depending on cell sizes. For ordinal or non-normally distributed variables, non-parametric approaches will be applied.

The surveys for different stakeholder groups include a limited number of identical or comparable questions. Where sufficient data are available, potential differences in perspectives between stakeholder groups will be explored through comparative analyses. Given the relatively small and potentially uneven group sizes, and the possibility that variables are not normally distributed, non-parametric statistical methods will be used. For comparisons between two independent groups, the Mann–Whitney U test will be applied, and for comparisons involving more than two groups, the Kruskal–Wallis test will be used. For categorical variables, group differences will be examined using the Chi-square test or Fisher’s exact test, particularly when expected cell counts are small.

Given the relatively small sample size, missing data will be handled using complete case analysis (listwise deletion). The number of respondents for each question will be reported. Potential bias due to missing data and non-response will be considered where feasible. Given the exploratory nature of the analyses, the results will be interpreted with caution, with emphasis placed on identifying patterns and generating insights in relation to the study aims.

#### 2.5.1 Data integration strategy

Along with the integrated mixed methods study design, a theory-oriented multi-perspective data integration strategy (45) is applied in the present study. Following Bazeley (61), integration is understood as an iterative process in which qualitative and quantitative data and analyses are combined to become interdependent in addressing the research aims. Although, the degree of integration depends on the research question being answered, i.e., some questions integrate multiple data sources, while others can be answered with a single data type. In the dataset, different materials−whether qualitative or quantitative, regardless of the data source−have equal status in the analysis.

An abductive analytic strategy is applied in this study, in which iterative movement between empirical data and TMFs is used to generate the most plausible explanations for the observed phenomena (50, 62). In terms of interpretation and reasoning, the core feature in the iterative analysis process is interaction within the dataset. The analysis, especially in terms of meta-inference, will be informed by TMFs, with additional patterns and themes being allowed to emerge inductively from the data, examining manifest content of the data. Abductive reasoning supports meta-inference by iteratively integrating qualitative and quantitative findings to generate the most plausible, overarching explanations that reconcile patterns across datasets. Critical awareness of assumptions, perspectives, and potential influences on interpretation will be required throughout the analysis process.

Data triangulation has multiple, although complementary, purposes in this study. First, data triangulation is used for convergence and corroboration to scrutinize whether results based on qualitative and quantitative data analysis or from different data sources point to the same conclusions. Secondly, it is used to show how different methods or data sources illuminate diverse dimensions or contradictions of the studied phenomenon. All in all, through triangulation, methodological rigor and inferential validity of the study are strengthened (44, 63).

As the study is conducted by a research team of three researchers from different disciplines, researcher triangulation is used to reduce bias in observations or interpretations that may arise from an individual researcher (64). However, in this study, the most important dimension, because of human interaction and teamwork, is the enhancement of the analysis process and interpretation.

## 3 Discussion

This protocol outlines how vocational rehabilitation job coaching is examined using a mixed methods implementation study design. The IPS model and its approach are gradually becoming a full-scale strategy for promoting inclusive and sustainable employment for people with serious health and psychosocial challenges and may even form the basis for future active labor market policy in Europe (65). The study addresses a timely research need by examining the implementation of IPS-based personalized vocational rehabilitation by generating new knowledge on the application of the IPS-based job coaching to a broader target group of working-age individuals whose ability to work or study has been substantially reduced. In particular, there is a significant knowledge gap concerning the role of supervisors and workplaces in this type of personalized vocational rehabilitation.

The mixed methods study design, incorporating a dataset compiled from multiple data sources, enables a comprehensive exploration of the phenomenon. It also helps to mitigate potential risks related to data collection. Because the service is newly launched, the number of clients may initially be lower than expected, which could affect the number of survey respondents. Applying multiple implementation science TMFs (Proctor’s framework, the CFIR, TDF, and NPT) enables a robust and conceptually grounded approach to understanding the complex aims, mechanisms, and contextual contingencies that shape implementation processes. Each of these TMFs contributes a distinct analytical lens: Proctor’s framework clarifies and operationalizes implementation outcomes; CFIR provides a comprehensive structure for assessing multilevel determinants; TDF offers a theory-informed understanding of behavioral influences; and NPT captures the social mechanisms through which new practices become embedded in everyday work.

Used together, these TMFs offer complementary insights that strengthen interpretative depth and enhance the explanatory value of the findings. This approach also promotes methodological transparency and rigor and facilitates comparison with prior research that has applied similar frameworks. Moreover, the use of multiple TMFs helps ensure that the knowledge generated is both contextually grounded and transferable to related settings, addressing the central challenges of implementation research, where variation across contexts and systems often complicates synthesis and generalization.

## 4 Ethics and dissemination

This study was approved by the Ethics committee of the Social Insurance Institution of Finland (Kela, code number KELA/3998/2025). Registration was made in Open Science Framework on Feb 18, 2026 and up-dated on May 21, 2026 (ID: https://doi.org/10.17605/OSF.IO/N63H5). There are no articles or reports relating to the study that have been published or are under review.

Kela’s core mission is to implement Finland’s social security system by providing benefits and services—such as rehabilitation—and by producing knowledge to support its development and decision-making. In this study, the Kela organization has multiple roles: service funder and administrator (including rehabilitation claims processing), scientific researcher (including funding), and ethics committee (reviewing non-medical research based on guidelines drawn up by the Finnish National Board on Research Integrity).

This study is carried out by Kela’s Research Unit, an independent agency conducting scientific research. The statutory basis of Kela’s research is defined in the Act on the Social Insurance Institution (66), according to which Kela is responsible for conducting and monitoring research related to social security to support its implementation and development. Accordingly, the present study is funded by Kela. Kela as a rehabilitation service administrator, has no role in the study design, data collection, analysis, interpretation of the data, or writing of the manuscript.

The data controller of the study is Kela. A data protection notice for scientific research regarding the processing of personal data has been prepared and is available in Finnish at internet the page of the study (67). The notice provides the information required to be given to participants when personal data are processed in scientific research, in accordance with the European Union General Data Protection Regulation (68). Personal data refer to any information that can directly or indirectly identify an individual, for example by combining data elements that enable identification. A data protection impact assessment has been conducted for the study. Study documents will be destroyed no later than 31 December 2030 and will not be retained for future research.

The study involves the processing of personal data collected through surveys and interviews. The data are processed in a pseudonymized form. All participants receive an information sheet that explains the purpose and procedures of the study, as well as the voluntary nature of participation and the confidentiality of the data. Informed consent is requested from all study participants.

For online surveys, informed consent is requested on the front page of the questionnaire. In interviews, informed consent is requested orally and recorded as part of the audio recording. No direct personal data will be collected during the interviews, but participants may provide information about workplaces, regions, service use, health, and work circumstances that could enable direct or indirect identification; therefore, interview data will be pseudonymized to reduce identifiability. The results of the study are reported in a way that no individual person can be identified. The collected research data will be used solely for the purpose specified in the research plan. Only the researchers have access rights to the research data. The researchers are bound by Kela’s confidentiality agreement.

The results of the study will be published as peer-reviewed articles and presented at national and international rehabilitation and implementation science conferences. A policy paper addressed specifically to policymakers, along with guidelines for rehabilitation practitioners, will be published as a summary of the main findings to promote the implementation and normalization of personalized services in vocational rehabilitation. Progress of the study will be communicated interactively through social media, for example LinkedIn.
